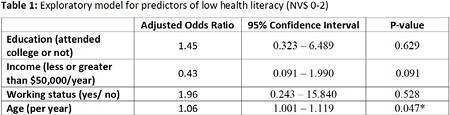# 736 Health Literacy in Patients with Burn Injury

**DOI:** 10.1093/jbcr/irae036.279

**Published:** 2024-04-17

**Authors:** Alexander Morzycki, Emily Eschelbach, Ayana Sharma, Jamie Oh, Adrian Battiston, Joshua N Wong, Tam N Pham

**Affiliations:** University of Washington, Harborview Burn Centre, Edmonton, AB; University of Washington, Harborview Burn Centre, Seattle, WA; University of Washington, Harborview Burn Centre, Seattle, WA; University of Washington, Seattle, WA; University of Alberta Burn Centre, Edmonton, AB; University of Washington, Harborview Burn Centre, Edmonton, AB; University of Washington, Harborview Burn Centre, Seattle, WA; University of Washington, Harborview Burn Centre, Seattle, WA; University of Washington, Seattle, WA; University of Alberta Burn Centre, Edmonton, AB; University of Washington, Harborview Burn Centre, Edmonton, AB; University of Washington, Harborview Burn Centre, Seattle, WA; University of Washington, Harborview Burn Centre, Seattle, WA; University of Washington, Seattle, WA; University of Alberta Burn Centre, Edmonton, AB; University of Washington, Harborview Burn Centre, Edmonton, AB; University of Washington, Harborview Burn Centre, Seattle, WA; University of Washington, Harborview Burn Centre, Seattle, WA; University of Washington, Seattle, WA; University of Alberta Burn Centre, Edmonton, AB; University of Washington, Harborview Burn Centre, Edmonton, AB; University of Washington, Harborview Burn Centre, Seattle, WA; University of Washington, Harborview Burn Centre, Seattle, WA; University of Washington, Seattle, WA; University of Alberta Burn Centre, Edmonton, AB; University of Washington, Harborview Burn Centre, Edmonton, AB; University of Washington, Harborview Burn Centre, Seattle, WA; University of Washington, Harborview Burn Centre, Seattle, WA; University of Washington, Seattle, WA; University of Alberta Burn Centre, Edmonton, AB; University of Washington, Harborview Burn Centre, Edmonton, AB; University of Washington, Harborview Burn Centre, Seattle, WA; University of Washington, Harborview Burn Centre, Seattle, WA; University of Washington, Seattle, WA; University of Alberta Burn Centre, Edmonton, AB

## Abstract

**Introduction:**

Burn injuries occur with a disproportionately higher frequency in vulnerable groups. Their health literacy (HL), defined as the way patients obtain, process, and understand health information, may be an important factor that influences access, care, and recovery after burns. This study aims to determine the current levels of health literacy and variables associated with poor health literacy for outpatients treated at two regional burn centres.

**Methods:**

All eligible patients presenting to two tertiary outpatient burn centers were asked to participate. The Newest Vital Sign health literacy assessment tool and a sociodemographic survey were administered. The NVS scores were divided into poor (0-2), moderate (3-4), and adequate (5-6) health literacy. We first compared health literacy rates between sites using non-parametric Chi-squared test. We then developed a multivariable regression model using a stepwise construction with poor health literacy as our dependent variable. Included in the exploratory model were income, education level, working status, and age.

**Results:**

Forty-nine patients with a mean age of 46.6 years (SD=17.4) were included; 57% (29/49) of patients identified as male. Most of the burn injuries (78%, 38/49) were less than 10% total body surface area. HL rates were poor, moderate, and adequate in 35% (17/49), 18% (9/29), and 47% (23/29), respectively. There were no differences in health literacy between the 2 sites. Age was the only independent predictor of poor health literacy in burn patients, with advanced age being associated with worse health literacy (95% CI: 1.001-1.119, p< 0.05). Income, education level and working status did not independently predict health literacy rates (Table 1).

**Conclusions:**

More than half of patients treated at burn outpatient clinics have only poor or moderate health literacy. This study further suggests that older adults may be most at risk. This trend is consistent across two regional burn centres. This study highlights the importance of health literacy among patients with burn injury and stresses the importance of personalized and literacy-adjusted discussions surrounding care. Our next project will address the impact of health literacy on adherence to recommended therapies.

**Applicability of Research to Practice:**

Poor health literacy is a barrier to obtaining burn care and is an important social determinant of health. We should identify factors associated with poor health literacy among patients with burn injury and improve the delivery of health information.